# Anatomy, biomechanics, and clinical advances of proximal tibiofibular joint in pain management

**DOI:** 10.3389/fsurg.2025.1604538

**Published:** 2025-07-17

**Authors:** Tianjun Zhai, Fengyan Jiang, Wei Feng, Zhaohui Chen

**Affiliations:** ^1^ School of Acupuncture and Tuina (School of Rehabilitation Medicine), Anhui University of Chinese Medicine, Hefei, China; ^2^ Chinese Medicine Department, Hangzhou West Lake District Beishan Street Community Health Service Center, Hangzhou, Zhejiang, China; ^3^ Chinese Medicine Department, The Second Rehabilitation Hospital of Shanghai, Shanghai, China

**Keywords:** proximal tibiofibular joint (PTFJ), biomechanics, osteoarthritis, proximal fibular osteotomy, synovial joint disorders

## Abstract

This article presents a descriptive review focused on the proximal tibiofibular joint (PTFJ), a synovial plane joint located posterolaterally beneath the lateral tibial plateau. The PTFJ facilitates axial load transmission, allows subtle tibiofibular motion, and works in conjunction with the distal syndesmosis to preserve ankle and knee function. Dysfunction—resulting from anatomical variation, trauma, instability, or degeneration—can lead to pain, mobility impairment, and nerve compression. Clinical conditions such as osteoarthritis and peroneal nerve entrapment are increasingly linked to PTFJ pathology. Novel therapies like proximal fibular osteotomy (PFO) have shown promise in treating medial compartment knee osteoarthritis based on the uneven settling theory. Diagnosis involves multimodal imaging, and management spans conservative, interventional, and surgical approaches. A comprehensive understanding of the PTFJ supports accurate diagnosis and the development of effective treatment strategies.

## 1 Introduction

The PTFJ is a small synovial articulation located just below the knee, connecting the proximal ends of the tibia and fibula (1). Despite its relatively modest size, the PTFJ significantly contributes to biomechanical integrity and stability of the lower limb. Historically underestimated, the PTFJ's role in gait and load distribution has gained recognition, highlighting its clinical relevance in various lower limb pathologies.

Functional abnormalities including instability, anatomical variations, or degenerative changes—are often implicated in chronic pain syndromes that pose diagnostic and therapeutic challenges. Pathologies at the PTFJ disrupt the kinetic chain and weight distribution, potentially exacerbating conditions in adjacent knee and ankle joints.

As a descriptive review, this article synthesizes current evidence regarding the anatomical and biomechanical characteristics of the PTFJ and explores its clinical implications in pain management. The goal is to enhance diagnostic accuracy and guide the development of effective therapeutic strategies.

## 2 Anatomy of the PTFJ

The articular surfaces of PTFJ are covered with smooth hyaline cartilage, and the joint capsule tightly envelops the articulation, containing a small amount of synovial fluid to facilitate lubrication during movement. Its stability is supported by the anterior and posterior superior tibiofibular ligaments, which limit excessive tibiofibular translation (2).

**Figure 1:**
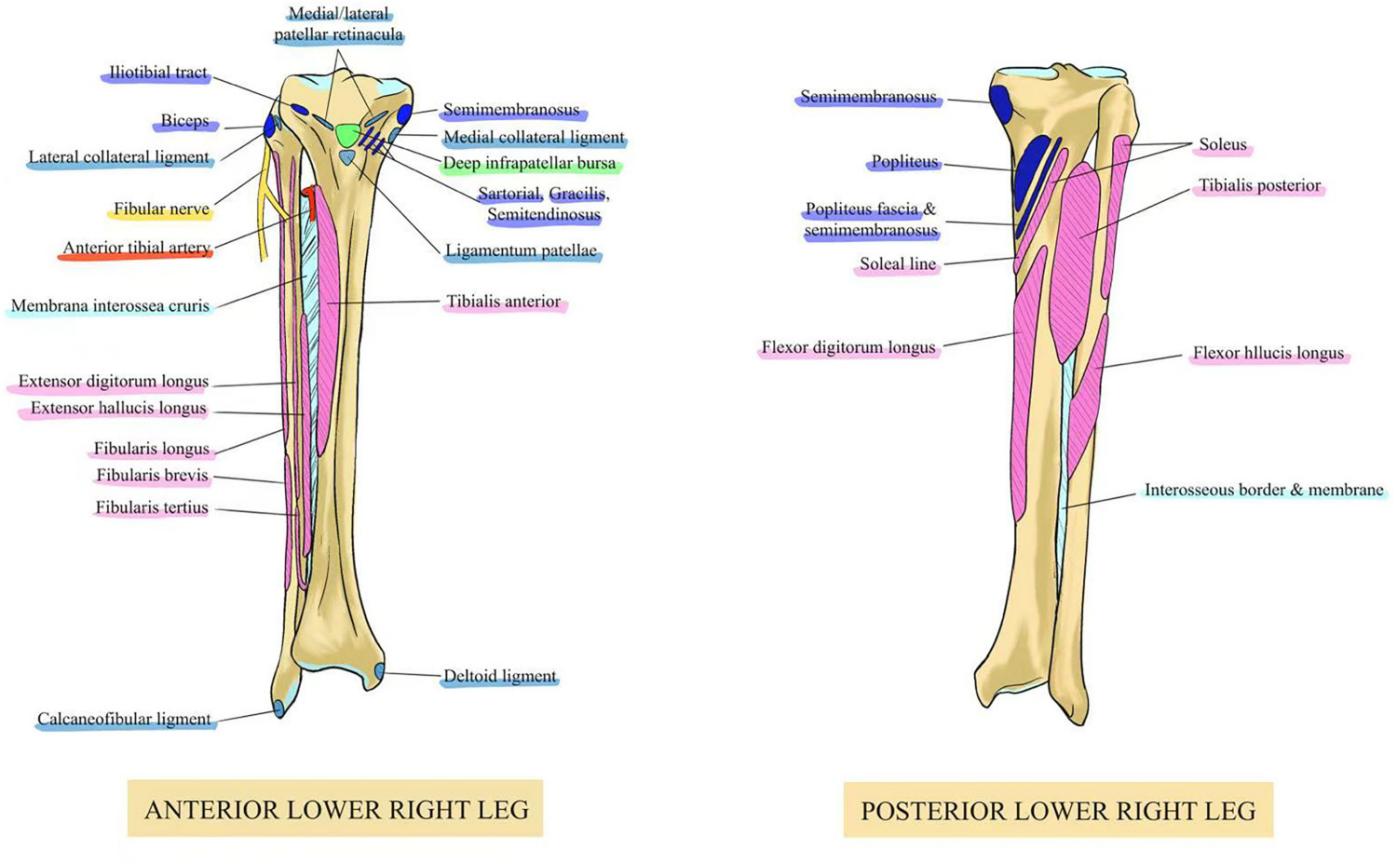
Key anatomical structures around the PTFJ.

Several clinically significant anatomical structures are located around the fibula. The surrounding musculature includes the fibularis (peroneus) longus, brevis, and tertius on the anterolateral side, and the biceps femoris, soleus, and tibialis posterior on the posterior aspect (Figure 1).

The fibularis longus originates from the fibular head and upper lateral shaft, and inserts onto the medial cuneiform and the base of the first metatarsal on the plantar surface of the foot. It contributes to ankle plantarflexion, foot eversion, and maintenance of the transverse arch. The fibularis (peroneus) tertius arises from the distal anterior surface of the fibula and the interosseous membrane, and inserts onto the dorsal surface of the base of the fifth metatarsal. It primarily facilitates ankle dorsiflexion and assists in foot eversion. Posteriorly, the biceps femoris muscle, originating from the ischial tuberosity and inserting into the posterolateral fibular head, is involved in knee flexion and external rotation of the leg. The soleus muscle, originating from the posterior tibia and fibula and inserting into the calcaneus, plays a critical role in plantarflexion during gait propulsion. The tibialis posterior, which also arises from the posterior tibia and fibula and inserts into the navicular and cuneiform bones of the foot, assists in plantarflexion, inversion, and arch stabilization. These muscle groups act in coordination to provide both stability and mobility to the ankle-foot complex, ensuring efficient gait mechanics and lower limb function (3). Neurologically, the PTFJ is in close proximity to several important nerve structures, most notably the common peroneal (fibular) nerve, tibial nerve, and the superficial and deep branches of the peroneal nerve. The common peroneal nerve courses around the posterior fibular head, making it vulnerable to compression from joint pathology. Its branches—the superficial and deep peroneal nerves—innervate key muscles of the lateral and anterior compartments (4). Understanding these neuromuscular relationships is crucial for diagnosing PTFJ-related dysfunction.

## 3 Biomechanical characteristics of the PTFJ

From a biomechanical perspective, the PTFJ plays a pivotal role in maintaining the functional stability of the lower limb. The PTFJ transmits approximately 16% of axial load during ankle dorsiflexion (5). Although its motion is subtle, the joint permits slight translation and rotation between the tibia and fibula, enabling essential adaptations to dynamic activities such as walking, running, or jumping. It works in tandem with the distal tibiofibular syndesmosis, ensuring coordinated movement and pressure distribution at the ankle mortise (6). This synergy maintains ankle congruity and protects against overloading. When PTFJ function is impaired—due to subluxation, ligament injury, or degeneration—it can lead to altered joint mechanics, pathological stress redistribution, and secondary complications such as cartilage wear, synovial inflammation, and instability-related pain (7).

Accordingly, a comprehensive understanding of the anatomical and biomechanical features of the PTFJ is essential for clinicians to accurately evaluate joint function, diagnose related disorders, and formulate targeted therapeutic interventions.

## 4 Clinical mechanisms of PTFJ-related pain

### 4.1 Anatomical variants and biomechanical abnormalities

Anatomical variations and biomechanical abnormalities of the PTFJ significantly affect joint stability and function. Congenital joint laxity or subluxation may lead to dynamic instability, manifesting as pain and functional impairment during movement. Studies have shown that PTFJ instability can arise from traumatic dislocation, chronic or recurrent subluxation, or non-traumatic joint displacement, with generalized ligamentous laxity being a major contributing factor in the non-traumatic cases (8). Furthermore, the morphology of the articular surfaces is closely linked to the development of degenerative changes. Obliquely oriented joint surfaces are associated with smaller contact areas, resulting in increased pressure per unit area, which may accelerate cartilage wear and osteoarthritic changes (9).

### 4.2 Degenerative disorders

PTFJ osteoarthritis (PTFJ-OA) is a relatively rare but clinically relevant degenerative condition, most commonly seen in elderly patients or those with prior joint trauma. Hallmark pathological features include joint space narrowing and osteophyte formation, leading to pain and restricted joint mobility. Clinically, patients often present with localized pain that may radiate to the lateral calf or ankle, affecting daily activities. Additional symptoms include joint stiffness, tenderness, and decreased flexibility, which are typically exacerbated after prolonged inactivity or in the early morning hours (10). These features resemble osteoarthritic changes in other joints and may significantly impair joint function and quality of life.

### 4.3 Trauma and inflammation

Acute trauma, particularly inversion injuries of the ankle, can generate strong tensile forces on the fibula, displacing the fibular head posterolaterally and leading to PTFJ subluxation or dynamic instability. Clinically, patients present with sharp localized pain, swelling, and tenderness, and in severe cases, impaired mobility. Repetitive or unresolved trauma may lead to capsular laxity and ligament weakening, exacerbating instability and predisposing the joint to chronic pain and functional deterioration (11). Additionally, chronic inflammatory conditions such as rheumatoid arthritis (RA) may involve the PTFJ. When affected, the joint displays synovitis, joint swelling, cartilage erosion, and bony destruction, leading to structural deformation, chronic pain, and impaired limb function.

### 4.4 Nerve entrapment

The common peroneal nerve courses superficially around the posterior margin of the PTFJ, making it vulnerable to compression or injury in the setting of joint pathology. In both acute and chronic injuries or degenerative changes, joint effusion or synovial cyst formation (e.g., Baker's cysts or synovial ganglia) can exert direct or indirect pressure on the nerve (12). This results in neurological symptoms such as numbness, paresthesia in the lateral calf, dorsum of the foot, and toes. In more severe cases, patients may experience motor deficits including weakness of the lateral compartment musculature and dorsiflexion impairment, leading to foot drop and gait disturbances. Prolonged compression without timely intervention may result in irreversible nerve damage (13).

### 4.5 Uneven settling theory

The “uneven settling” theory, an emerging biomechanical hypothesis, proposes that asymmetrical anatomical and mechanical load distribution among the femur, tibia, and fibula during weight-bearing may lead to functional disorders and pain in the lower extremity (14). Traditionally, the knee joint has been considered as comprising the tibiofemoral and patellofemoral components, while neglecting the biomechanical influence of the PTFJ. Despite its slender appearance, the fibula plays an integral role by articulating proximally at the PTFJ and distally contributing to ankle joint stability.

**Figure 2:**
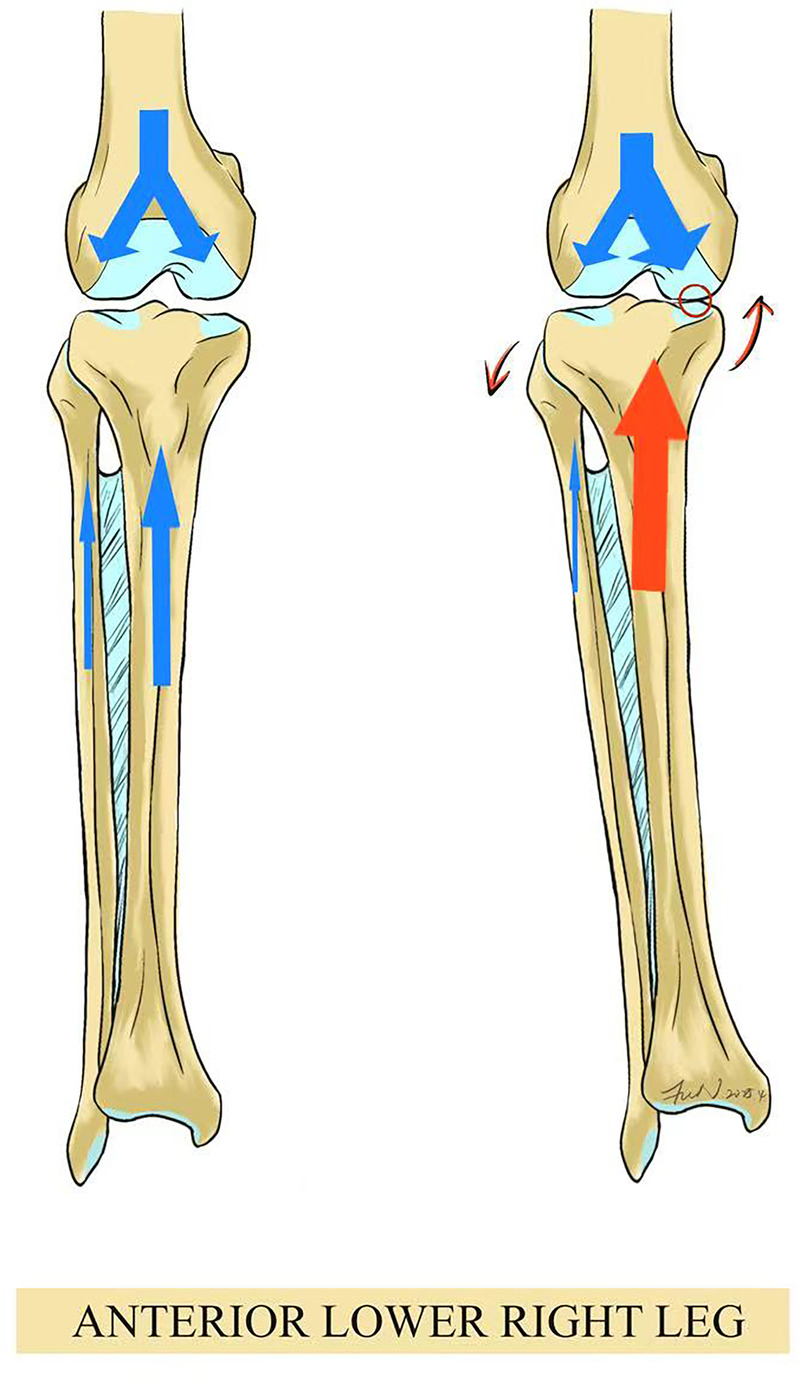
Uneven tibiofibular settling–induced medial knee osteoarthritis due to PTFJ dysfunction.

When PTFJ function is compromised—such as through subluxation or instability—this may disrupt the load-sharing relationship between the tibia and fibula. A downward shift of the fibula may fail to support the lateral tibial condyle adequately, causing the lateral tibial plateau to descend and the medial plateau to ascend, ultimately resulting in medial compartment narrowing and asymmetric osteoarthritis. This uneven stress distribution may promote cartilage wear, joint degeneration, and global biomechanical imbalance of the lower extremity (Figure 2) (15). Thus, PTFJ pathologies should not be overlooked in clinical settings, as aberrant settling patterns may represent a key mechanism underlying knee pain or broader joint dysfunction.

## 5 Recent advances in clinical applications of the PTFJ

### 5.1 Knee: proximal fibular osteotomy (PFO) and the uneven settling theory

**Figure 3:**
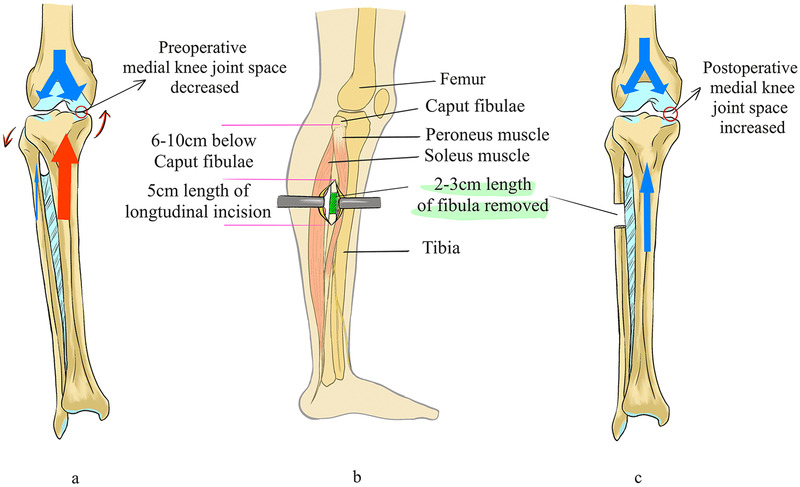
Illustration of PFO technique and its biomechanical impact on medial knee compartment [(a) preoperative joint stress concentration medially; (b) surgical site and fibula resection zone; (c) postoperative redistribution of load with increased medial joint space].

In recent years, proximal fibular osteotomy (PFO) has emerged as a novel surgical approach in the treatment of medial compartment knee osteoarthritis (KOA). Based on the “uneven settling” theory, this technique posits that excessive mechanical loading of the medial knee compartment is a primary factor driving KOA progression. By resecting the proximal segment of the fibula, PFO aims to redistribute joint loading, alleviate medial compartment pressure (Figure 3), and ultimately relieve pain while improving joint function (16).

Clinical studies have demonstrated promising outcomes. For example, a prospective study evaluating 21 patients undergoing PFO reported significant improvements within 12 months postoperatively: The visual analog scale (VAS) for pain decreased from 7.86 to 3.78. The American Knee Society Score (AKSS) improved from 56.49 to 72.71, and the functional AKSS from 48.24 to 71.46. The medial-lateral (ML) joint space ratio increased from 0.33 to 0.51, indicating improved load balance across the joint (17). Systematic reviews have further supported PFO's effectiveness in pain relief, functional restoration, and correction of varus deformity. However, the procedure remains somewhat controversial. Some researchers argue that the long-term efficacy and impact on disease progression require further verification. In addition, complications such as common peroneal nerve injury during surgery necessitate careful intraoperative technique (18).

Most existing studies are prospective case series with limited sample sizes and short follow-up durations, lacking support from randomized controlled trials (RCTs). For example, a prospective study conducted by Wang et al. reported improvements in pain and functional scores after PFO; however, the study did not include a control group and had a follow-up period of only 12 months, which limits the generalizability of its conclusions (19). Moreover, there is no consensus regarding the biomechanical mechanisms of PFO. Proposed theories include “uneven settling,” the “excessive cortical support theory,” and the “sliding phenomenon,” but none have been validated by high-quality studies (20). Therefore, despite the promising preliminary findings, the long-term efficacy and safety of PFO still require further confirmation through large-scale, randomized controlled trials.

Compared to established treatments like high tibial osteotomy (HTO), pharmacologic therapies, and physical rehabilitation, PFO's long-term outcomes and comparative effectiveness are still unclear. Therefore, larger randomized controlled trials are necessary to confirm its safety, durability, and clinical positioning in the treatment hierarchy of medial knee osteoarthritis.

In conclusion, PFO presents a potentially effective alternative in the treatment of medial KOA, with early studies indicating pain reduction and functional benefits. Nevertheless, more robust randomized controlled trials and long-term follow-up studies are needed to validate its safety and durability.

### 5.2 Lower leg: clinical manifestations of PTFJ dysfunction

Dysfunction of the PTFJ—such as instability, subluxation, chronic inflammation, or degeneration—can disrupt the biomechanical equilibrium between the tibia and fibula, resulting in pain, muscle spasms, and functional impairment in the lower leg. Patients typically present with persistent or load-dependent aching pain along the lateral leg, particularly aggravated by stair climbing, squatting, or prolonged ambulation (21). Due to the anatomical proximity of the PTFJ to surrounding muscles, tendons, and nerves, some patients also experience weakness in the lateral leg musculature. In advanced or prolonged cases, joint effusion or synovial cysts may compress the common peroneal nerve, leading to paresthesia or numbness over the lateral leg and dorsum of the foot. In severe instances, dorsiflexion weakness or foot drop may occur, profoundly impairing gait and mobility. If left unrecognized and untreated, PTFJ dysfunction can compromise the mechanical coordination of the distal tibiofibular articulation, accelerating degenerative changes in both the ankle and knee, and further contributing to widespread lower limb biomechanical disorders (22). Therefore, early identification, radiographic assessment, and targeted treatment of PTFJ pathology are crucial for restoring leg function and preserving quality of life.

### 5.3 Ankle: influence of PTFJ stability on ankle biomechanics

Although anatomically located proximally, the stability of the PTFJ is fundamental to the functional biomechanics of the ankle joint. The fibula connects to the tibia through the PTFJ, forming a dynamic load-transmitting structure. During lower limb movements—especially ankle dorsiflexion and plantarflexion—the fibula must undergo subtle but essential translational and rotational adjustments to preserve ankle stability and load distribution (23). Injuries or degenerative changes affecting the PTFJ—such as from acute inversion sprains, chronic ligament laxity, or osteoarthritis—may alter the spatial relationship between the tibia and fibula, leading to malalignment or displacement of the fibular head. This misalignment can destabilize the distal tibiofibular syndesmosis and ankle joint, contributing to chronic pain, instability, or even post-traumatic arthritis (24). Additionally, aberrant fibular motion caused by PTFJ dysfunction can compress the common peroneal nerve, resulting in sensory disturbances in the dorsum of the foot and lateral leg, along with dorsiflexion weakness, foot drop, and impaired gait. Such conditions can severely compromise physical activity and quality of life if not promptly addressed (25).

### 5.4 Interdependence of the lower limb kinetic chain

The PTFJ plays an integral role in the lower limb kinetic chain and should not be evaluated in isolation. Dysfunction of the PTFJ—whether due to instability, subluxation, or degenerative changes—can disrupt normal force transmission and segmental coordination between the ankle, knee, and hip joints.

For example, PTFJ instability may lead to excessive external rotation of the tibia, contributing to altered patellofemoral tracking, medial knee overload, and varus malalignment (26). Similarly, abnormal fibular movement can interfere with distal tibiofibular syndesmosis tension, impairing ankle stability during stance and push-off phases of gait (27). Over time, such biomechanical disruptions may provoke compensatory movements at the hip, such as increased internal rotation or pelvic tilt, potentially resulting in gluteal overuse or hip impingement syndromes.

Moreover, recent evidence suggests that lower limb dysfunction in chronic ankle instability may be associated with proximal compensatory changes. A study demonstrated that individuals with CAI exhibit abnormal isometric strength in the knee extensors and flexors, indicating kinetic chain involvement beyond the ankle itself (28). This supports the hypothesis that proximal joint alterations, including PTFJ pathology, may be biomechanically relevant in populations with chronic ankle disorders.

Clinically, this reinforces the importance of assessing the entire kinetic chain when managing lateral knee or leg pain. Functional tests (e.g., single-leg squat, gait analysis) and kinetic imaging may help identify upstream or downstream contributors to PTFJ-related pathology. Therapeutic approaches—whether conservative, interventional, or surgical—should therefore be individualized with consideration of the global biomechanical context.

## 6 Imaging and diagnostic approaches

Accurate diagnosis of proximal tibiofibular joint (PTFJ) disorders requires a multimodal imaging approach tailored to clinical suspicion and symptomatology.

### 6.1 Magnetic resonance imaging (MRI)

MRI is considered the most sensitive modality for evaluating soft tissue abnormalities around the PTFJ. It can detect joint effusion, synovitis, capsular thickening, ligamentous injuries, and adjacent muscle edema. Moreover, MRI is especially useful in identifying nerve compression from cysts or inflammatory lesions, such as common peroneal nerve entrapment (29).

### 6.2 Ultrasound

Ultrasound offers a dynamic, real-time, and cost-effective tool for evaluating superficial structures of the PTFJ. It allows visualization of joint effusion, synovial cysts, and ligament laxity during movement. Additionally, ultrasound-guided injections and interventions around the PTFJ can be safely and precisely performed under imaging guidance (30).

### 6.3 Weight-bearing computed tomography (WBCT)

WBCT provides high-resolution imaging of bony morphology under physiological load. It is particularly useful in detecting subtle joint incongruities, subluxation, or osteoarthritic changes not evident in standard radiographs. WBCT may also be helpful in surgical planning, especially in patients considered for proximal fibular osteotomy (31).

Integrating these imaging modalities helps clinicians achieve a more accurate and comprehensive understanding of PTFJ pathology, supporting better differential diagnosis and tailored treatment planning.

## 7 Treatment strategies for PTFJ-related disorders

Management of disorders involving the PTFJ includes conservative treatment, minimally invasive interventions, and surgical procedures.

### 7.1 Conservative management

Conservative treatment remains the first-line strategy for mild to moderate PTFJ disorders. Treatment options include nonsteroidal anti-inflammatory drugs (NSAIDs), physical therapy, activity modification, and Intra-articular corticosteroid injections, with the primary goals of relieving symptoms, improving joint function, and slowing disease progression. NSAIDs are commonly prescribed to control local inflammation and alleviate pain. However, long-term NSAID use may lead to gastrointestinal discomfort, mucosal injury, and increased cardiovascular risk. Thus, close monitoring and concomitant use of gastroprotective agents are recommended when necessary (32). Physical therapy should focus on strengthening peroneal and hamstring muscles, correcting gait abnormalities, and improving joint proprioception. Bracing or orthotics may be beneficial for patients with joint hypermobility or instability. Intra-articular corticosteroid injections are also widely used in clinical practice. Corticosteroids can effectively suppress local inflammation and reduce joint effusion, providing rapid symptomatic relief in the short term (33, 34). Nevertheless, the long-term efficacy of corticosteroid therapy remains controversial. Frequent injections may damage articular cartilage, accelerate degeneration, and increase the risk of osteoporosis. As a result, it is advised that steroid injections be limited to no more than 3–4 times per year, and administered under specialist supervision (35). However, conservative approaches often yield limited success in cases with.

### 7.2 Interventional therapies

When conservative measures fail, interventional techniques can offer pain relief and functional improvement. Interventional techniques such as radiofrequency ablation (RFA) and platelet-rich plasma (PRP) injections have gained traction.

#### 7.2.1 RFA

RFA involves targeting sensory branches around the fibular head using fluoroscopic or ultrasound guidance. Pulsed or conventional RFA may be applied at 60–90°C for 90–120 s. Ideal candidates include patients with localized PTFJ pain who have failed conservative therapy and are not suitable for surgery. RFA is generally safe and well tolerated, with a low incidence of adverse events (36).

#### 7.2.2 PRP

PRP is typically administered as 3–5 ml intra-articularly under ultrasound guidance. Injection technique involves a lateral or posterolateral approach targeting the joint capsule or surrounding synovium. PRP is best suited for patients with early degenerative changes, chronic synovitis, or post-traumatic inflammation (37). Response rates vary, and repeated injections may be required every 6–12 months.

### 7.3 Surgical management

Surgical intervention is typically reserved for patients with severe degenerative disease or refractory pain that fails to respond to conservative and interventional therapies. Two main surgical options are:

#### 7.3.1 Arthrodesis (joint fusion)

This procedure is considered a definitive treatment for advanced PTFJ osteoarthritis or instability. Fusion of the proximal tibiofibular joint eliminates joint motion, significantly relieves pain, and improves mechanical stability. Clinical data suggest a fusion success rate >90%, with substantial pain reduction and enhanced quality of life postoperatively. However, the loss of joint motion may restrict lateral knee rotation and deep squatting, limiting certain functional activities (38).

#### 7.3.2 Fibular head resection

This technique involves partial or complete resection of the fibular head to relieve abnormal mechanical stress or excise painful lesions. While this can be effective in pain control, it carries the risk of distal fibular instability, which may in turn compromise ankle function and disrupt overall lower limb biomechanics. Thus, strict postoperative rehabilitation and long-term follow-up are crucial to monitor potential complications (39).

### 7.4 Clinical decision-making flowchart

**Figure 4:**
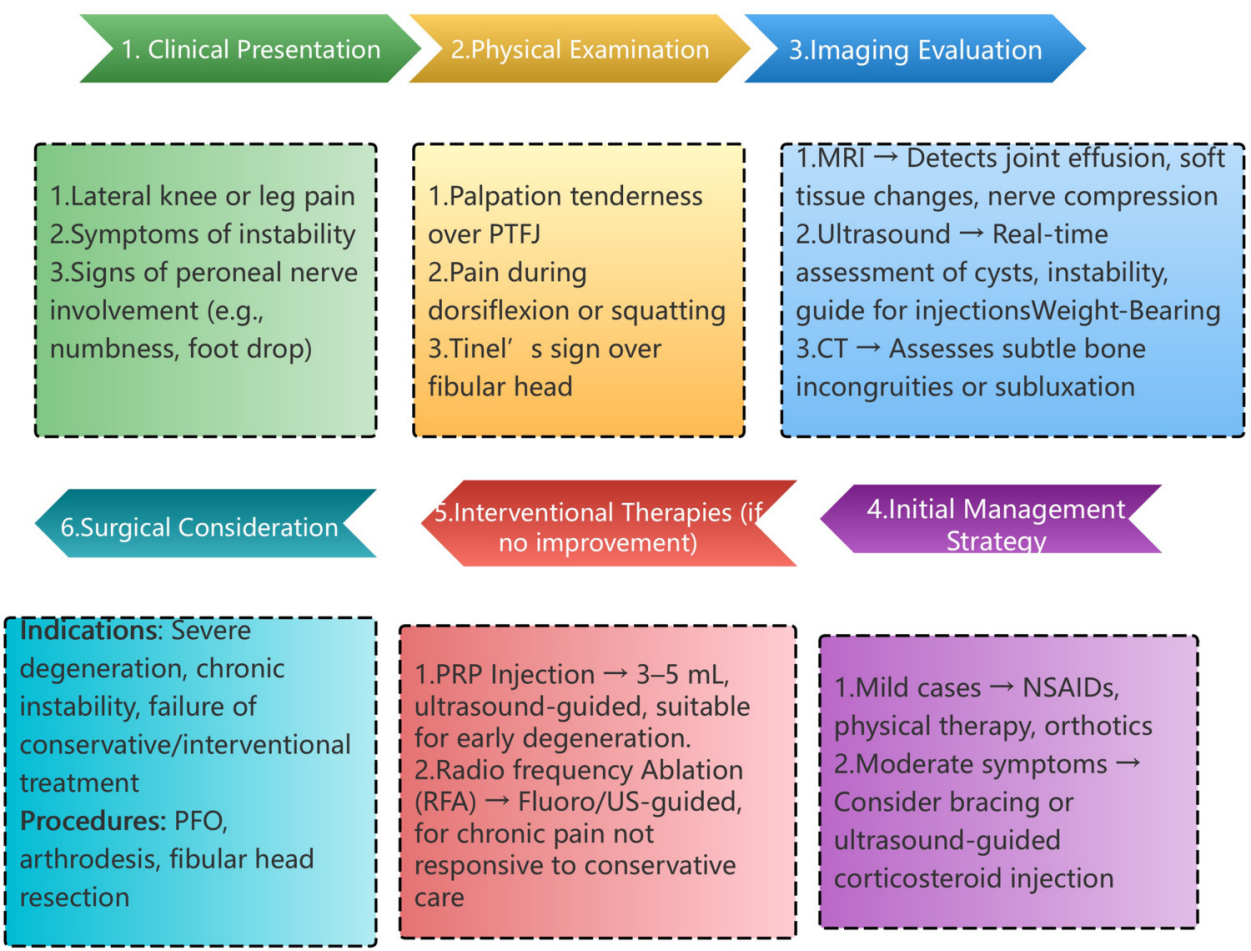
Diagnostic and treatment flowchart for PTFJ disorders.

To assist clinicians in selecting appropriate diagnostic and therapeutic pathways, we propose a practical algorithm (Figure 4), which considers symptom severity, imaging findings, and patient-specific factors to guide treatment selection.

### 7.5 Evidence strength and limitations

While this review compiles a wide range of studies on the anatomy, biomechanics, and clinical management of the PTFJ, it is important to critically evaluate the quality and strength of the evidence presented.

Most clinical studies related to PTFJ, particularly those concerning interventions like proximal fibular osteotomy (PFO), are small-scale prospective case series or observational cohorts. Although these studies offer valuable insights, they are limited by the absence of control groups, short follow-up durations, and lack of randomization. For example, several studies reporting positive outcomes after PFO lack comparator arms, making it difficult to isolate treatment effects from placebo or natural history. Similarly, data on platelet-rich plasma (PRP) and radiofrequency ablation (RFA) for PTFJ-related conditions are often extrapolated from general knee or ankle osteoarthritis studies, not specifically designed for the PTFJ.

High-quality randomized controlled trials (RCTs) focusing exclusively on PTFJ disorders remain scarce. Furthermore, biomechanical theories such as “uneven settling” have yet to be validated by large-scale experimental studies. Imaging studies, while advancing, are also predominantly descriptive or anatomical rather than diagnostic trials with standardized sensitivity or specificity reporting.

Therefore, although preliminary findings are promising, clinical application of these strategies should proceed cautiously, ideally within the framework of individualized assessment and guided by the best available—but limited—evidence. Continued efforts to develop PTFJ-specific RCTs and diagnostic accuracy studies are essential for evidence-based advancement in this field.

## 8 Conclusion

This review has examined the anatomy, biomechanics, and clinical significance of the proximal tibiofibular joint (PTFJ), emphasizing its pivotal role in maintaining lower limb stability and functional integrity. Special attention has been given to the clinical application of proximal fibular osteotomy (PFO), grounded in the uneven settling theory, for the treatment of medial compartment knee osteoarthritis. Although recent studies have provided valuable insights into PTFJ function and pathology, our current understanding remains incomplete. Future research should aim to explore the biomechanical behavior of the PTFJ under diverse conditions and validate the long-term efficacy and safety of novel therapeutic strategies such as PFO and PRP. In conclusion, this paper is a descriptive review that synthesizes current anatomical, biomechanical, and clinical evidence on the proximal tibiofibular joint (PTFJ). A deeper understanding of PTFJ pathology will enable clinicians to develop more effective diagnostic tools and individualized treatment protocols for improving patient outcomes.
